# Improving Morphological Quality and Uniformity of Hydrothermally Grown ZnO Nanowires by Surface Activation of Catalyst Layer

**DOI:** 10.1186/s11671-017-1838-x

**Published:** 2017-01-18

**Authors:** Gonzalo Murillo, Helena Lozano, Joana Cases-Utrera, Minbaek Lee, Jaume Esteve

**Affiliations:** 1 Department of Nano and Microsystems, Instituto de Microelectrónica de Barcelona (IMB-CNM, CSIC), 08193 Bellaterra, Spain; 2 Department of Physics, Inha University, Incheon, 22212 South Korea

**Keywords:** ZnO nanowires, Gold catalyst layers, Hydrothermal growth, Cyclic voltammetry, Electrochemistry

## Abstract

This paper presents a study about the dependence of the hydrothermal growth of ZnO nanowires (NWs) with the passivation level of the active surface of the Au catalyst layer. The hydrothermal method has many potential applications because of its low processing temperature, feasibility, and low cost. However, when a gold thin film is utilized as the seed material, the grown NWs often lack morphological homogeneity; their distribution is not uniform and the reproducibility of the growth is low. We hypothesize that the state or condition of the active surface of the Au catalyst layer has a critical effect on the uniformity of the NWs. Inspired by traditional electrochemistry experiments, in which Au electrodes are typically activated before the measurements, we demonstrate that such activation is a simple way to effectively assist and enhance NW growth. In addition, several cleaning processes are examined to find one that yields NWs with optimal quality, density, and vertical alignment. We find cyclic voltammetry measurements to be a reliable indicator of the seed-layer quality for subsequent NW growth. Therefore, we propose the use of this technique as a standard procedure prior to the hydrothermal synthesis of ZnO NWs to control the growth reproducibility and to allow high-yield wafer-level processing.

**Electronic supplementary material:**

The online version of this article (doi:10.1186/s11671-017-1838-x) contains supplementary material, which is available to authorized users.

## Background

In most environments regardless of human endeavors, it is not difficult to find sources of residual energy which are being usually wasted, e.g., light, heat, or motions. The conversion of such ambient energy into electricity is of great interest, so that energy harvesting research field has been drawing special attentions in past years [1, 2]. Aside from other energy sources, mechanical energy present in various forms such as vibrations, random motions, human movements, or noise. The most common transduction methods to convert this mechanical energy into electricity are electrostatic, electromagnetic, triboelectric, and piezoelectric. In particular, a piezoelectric material has the ability of creating an inherent electric field when strained (i.e., direct piezoelectric effect). Nowadays, there are several examples of commonly used piezoelectric materials such as AlN, PZT, ZnO, PVDF, or quartz. Recently, ZnO has become very popular due to the extensive diversity of nanostructures that can exhibit. In addition, this material has the property of being a semiconductor, piezoelectric, and direct bandgap material which makes possible a huge range of applications [3, 4]. One of the most useful nanostructures that can be utilized to generate energy is the nanowire (NW) because of the simple alignment of its crystal axis during the growth process [5, 6]. Power generators based on these nanostructures are commonly called nanogenerators, and they have the advantages of being more flexible and less sensitive to fracture than generators based on thin films [7]. It has already been demonstrated that a single ZnO NW can generate a piezoelectric potential along its length in response to a 5-nN force applied by the tip of an atomic force microscope (AFM) [8]. The energy output generated by one NW in one discharge event was calculated to be 0.05 fJ. The ZnO NWs can be grown via different methods and on various substrates, but a crystalline substrate with a similar lattice constant is the best choice to obtain aligned NWs with high quality [9, 10].

Currently, there are numerous bottom-up approaches to grow ZnO nanostructures, such as vapor–solid and vapor–liquid–solid processes or electrochemical deposition [6, 11, 12]. However, these methods require high temperatures and pressures, conductive substrates, or acid-resistant environments that make them difficult to be integrated with standard fabrication processes and future flexible electronics.

At the beginning, organometallic synthesis of ZnO nanoparticles in an alcoholic medium received wider acceptance and more attention because it offered faster nucleation and growth than in a water-based medium. However, there are several reports of hydrothermal synthesis in an aqueous medium in the literature. Baruwati et al. reported the aqueous synthesis of ZnO nanoparticles using zinc nitrate hexahydrate in an autoclave at 120 °C after adjusting the pH to 7.5 with ammonium hydroxide [13]. They obtained ZnO nanoparticles in powder form after washing and drying. Lu et al. successfully prepared crystalline ZnO powder through a hydrothermal process, using ammonia as the base source and Zn(NO_3_)_2_ as the Zn^2+^ ion source [14]. The effects of growth temperature and pH were studied, and their results showed that all of the zinc hydroxide precursors dissolved and formed a clear solution at pH > 11 to nucleate ZnO powder in a homogeneous solution. On the other hand, the zinc hydroxide precursors were partially dissolved at pH < 11, and the ZnO powder nucleated in a heterogeneous system with higher probability of nucleation.

In this work, we utilized a hydrothermal method to grow *c*-axis-aligned NWs due to the combination of being inexpensive, simple, and with a mild processing temperature. This method is based on a chemical reaction that takes place directly on a silicon substrate covered by an Au catalyst layer in presence of an aqueous nutrient solution of zinc nitrate hexahydrate (Zn(NO_3_)_2_·6H_2_O) and hexamethylenetetramine (HMTA) at mild temperatures (<80 °C) [15]. The growth time, processing temperature, and nutrient concentration have a direct influence on the resulting height and diameter of the growth NWs [16].

In the aqueous solution, Zn(NO_3_)_2_·6H_2_O provides Zn^2+^ ions required for the ZnO NWs, water provides O^2−^ ions, and the thermal degradation of HMTA allows the release of hydroxyl ions to form ZnO by reacting with Zn^2+^ ions. This process can be summarized by Eqs. 1, 2, and 3 as follows:

1 $$ {\left(\mathrm{C}{\mathrm{H}}_2\right)}_6{\mathrm{N}}_4+6{\mathrm{H}}_2\mathrm{O}\leftrightarrow 6\mathrm{HCHO}+4\mathrm{N}{\mathrm{H}}_3 $$

2 $$ \mathrm{N}{\mathrm{H}}_3+{\mathrm{H}}_2\mathrm{O}\leftrightarrow \mathrm{N}{{\mathrm{H}}^{+}}_4+\mathrm{O}{\mathrm{H}}^{-} $$

3 $$ 2\mathrm{O}{\mathrm{H}}^{-}+\mathrm{Z}{\mathrm{n}}^{2+}\to \mathrm{Z}\mathrm{n}\mathrm{O}\left(\mathrm{s}\right)+{\mathrm{H}}_2\mathrm{O} $$

Even though the precise role of HMTA during the ZnO nanowire growth is still uncertain, it seems to act as a weak base, which would slowly hydrolyze in the aqueous solution to gradually produce OH^−^. The role of HMTA during the ZnO nanowire growth seems to be related to its slow hydrolysis in the aqueous solution that gradually produces OH^−^. This is a key aspect in the synthesis process because if HMTA hydrolyzes too fast, many OH^−^ ions will be produced in a short period of time resulting in a premature ZnO precipitation.

The quality of the gold catalyst layer is an important parameter to achieve an adequate nucleation leading to the growth of well-oriented NWs with a homogeneous distribution. A non-cleanroom environment exposes the gold surface to numerous ambient contaminants that affect the binding kinetics, electrochemical effects, and surface passivation.

One of the most critical issues of this growth method is the poor reproducibility and homogeneity of the grown NWs. Similar substrates with identical catalyst layers and synthesis conditions can produce different growth results. We hypothesize that gold surface passivation or contamination is a major contributor to this variability. Inspired by traditional electrochemistry experiments in which gold electrodes are typically activated (i.e., via surface cleaning) before performing the electrochemical measurements, we have demonstrated that similar activation processes are also useful in assisting and enhancing the ZnO NW growth [17].

## Methods

In electrochemistry experiments, scientists typically activate gold electrodes before performing their measurements. Lee et al. presented several cleaning processes to activate gold electrodes [17]. In this section, we describe several experiments performed to demonstrate that the surface activation of the gold catalyst layer immediately before the NW growth favors ZnO nucleation on the gold surface and improves the reproducibility of the grown ZnO NWs.

### Chemicals and Samples

Potassium nitrate (98.5%), potassium ferricyanide (99%), potassium ferrocyanide (98.5%), NaOH (97%), KOH (90%), H_2_O_2_ (30%), zinc nitrate hexahydrate (98%), HMTA (99%), and nitric acid (65%) were purchased from Sigma-Aldrich, USA, and used as received. All solutions were prepared using deionized water with resistivity greater than 18 MΩ cm.

In order to obtain the samples used in the experiments, a catalyst layer of gold together with a chromium adhesion layer (50-nm Au/20-nm Cr) were deposited by electron-beam evaporation on top of an n-type silicon (100) wafer. Finally, the wafer was diced to obtain 6 mm × 6 mm chips. All the samples used for the activation experiments were stored in ambient condition for 1 year. Prior to any activation step, all the samples were sequentially cleaned by immersing them in acetone for 10 min, ethanol for 5 min, and IPA (isopropyl alcohol) for another 5 min, and finally, they were rinsed in deionized water and dried with N_2_.

### Activation Processes

Based on the findings of Lee et al., more promising activation processes have been tested to find the optimal procedure that maximizes the morphological quality, density, and vertical orientation of the grown NWs [17]. We focused on chemical-based activation processes as they will lead to an easier wafer-level processing in the future. An aqueous solution of NaOH at a concentration of 50 mM and another solution of KOH at the same concentration were prepared with deionized water. Afterwards, each solution was mixed with diluted H_2_O_2_ at two different solute/volume ratios, 1:2 and 1:3 (i.e., one part of diluted H_2_O_2_ for 2 and 3 parts of alkaline solution, respectively), to yield a total of four different alkaline activation solutions. Hereafter, they are referred to as H_2_O_2_:NaOH and H_2_O_2_:KOH, followed by the volume ratio in square brackets, e.g., H_2_O_2_:NaOH [1:2], H_2_O_2_:KOH [1:3]. Each alkaline solution was used to activate the gold layer’s surface for 5 or 10 min. An acid solution of HNO_3_ (0.1 M) was also used to activate the samples for 1, 3, or 10 min. The samples were exposed to ultrasonic waves during activation to avoid the formation of H_2_ bubbles on the gold surface. At the end of this step, each sample was immersed in deionized water to stop the activation reaction.

### Hydrothermal Growth of ZnO Nanowires

Once the Au catalyst layer on the Cr/Si substrate was activated, NWs were grown by the hydrothermal method in a raw to prevent any contamination. Zinc nitrate and HMTA were mixed to create an equimolar aqueous solution at 5 mM. Afterwards, every chip was placed in a wide-mouth jar containing the aqueous solution so that it floated on the surface with the catalyst layer facing down. The jar was then sealed and placed in a convection oven at 70 °C for 16 h. A graphical scheme of the chemical process taking place during this treatment is presented in Additional file 1: Figure S1. As the temperature increased, ZnO molecules slowly precipitated and started to form NWs via epitaxial growth, facilitated by a proper nucleation on the activated gold surface. Finally, to avoid stiction aggregation of the grown nanostructures from surface tension caused by solvent evaporation, the samples were dipped in ethanol, which has a lower surface tension than water, and dried at ambient temperature.

### Morphological Inspection

Scanning electron microscope (SEM) images of all the samples after the seed-layer activation and the NW growth were carefully examined to perform a qualitative analysis of the NW morphologies based on the different activation (i.e., cleaning) conditions. There, images were taken with an SEM AURIGA 40 (Carl Zeiss, Oberkochen, Germany) at 2 KeV and using a secondary-electron detector. Afterwards, the activated samples with better cleaning performance in terms of resulting NW quality were carefully analyzed by cyclic voltammetry to evaluate the activation of the gold catalyst layer, using it as an indicator of the seed-layer quality for subsequent NW growth. Also, AFM measurements in contact mode were performed to analyze the surface roughness, where a significant increase in roughness would indicate undesired excessive processing of activation treatments on the gold surface. An AFM Veeco Nanoscope Dimension 3100 (Digital Instruments-Veeco, Woodbury, NY, USA) was utilized.

### Electrochemical Characterization

**Fig. 1:**
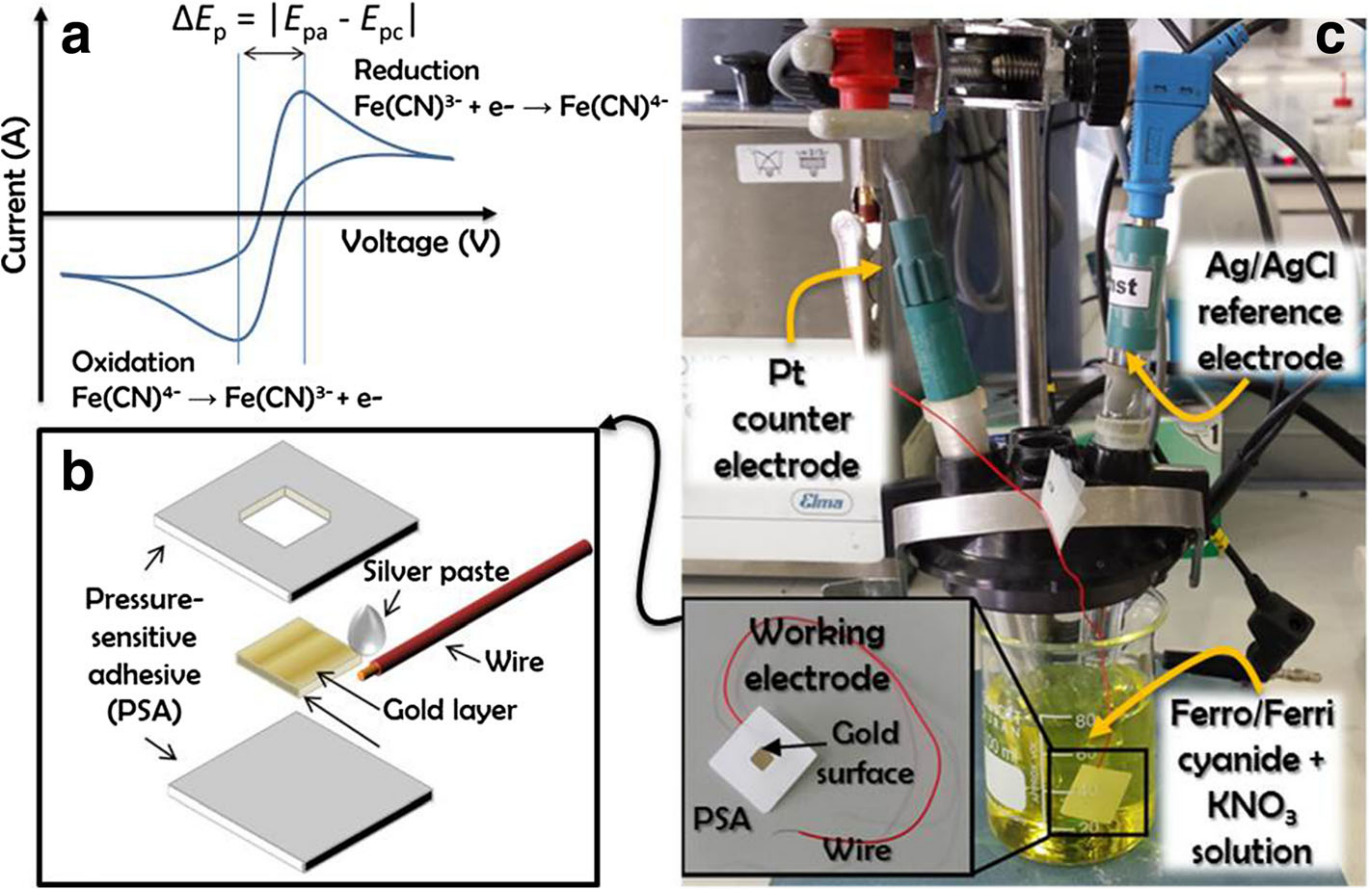
Characterization performed by cyclic voltammetry. **a** Typical cyclic voltammetry response. **b** Schematic drawing of the sample packaging to build the working electrodes. **c** The electrochemical setup used to measure cyclic voltammograms

Cyclic voltammetry (CV) was carried out in an Autolab PGStat10 potentiostat (Metrohm, The Netherlands) with a three-electrode configuration. A typical CV response that can be obtained with this equipment is shown in Fig. 1a, where Δ*E*_p_ is defined as the voltage difference between the oxidation and reduction peaks. To acquire this type of measurement, three different electrodes were utilized: a working electrode where oxidation and reduction take place, a reference electrode that works as the zero voltage level and maintains a constant potential, and a counter electrode that conducts electricity from the potentiostat signal source to the working electrode.

The Pt-ring counter electrode (6.0351.100, Metrohm) and Ag/AgCl reference electrode (6.0750.100, Metrohm) were commercially available and used for all the measurements. In order to fabricate the working electrode, each sample containing a catalyst layer was bonded to a wire with silver paint and electrically isolated by using a custom-made package consisting of pressure-sensitive adhesive (PSA), leaving only an exposed gold area of 4 mm × 4 mm, as shown in Fig. 1b. The measurements were performed with a potential sweep ranging from 0.10 to 0.45 V at a scan rate of 0.05 V s^−1^, and the electrochemical setup used to measure the cyclic voltammograms is shown in Fig. 1c. The three electrodes were immersed in the sample solution comprising a mixture of 20 mM ferro- and ferricyanide, [Fe(CN)_6_]^3−^ and [Fe(CN)_6_]^4−^, and an electrolytic solution of 0.5 M KNO_3_ in deionized water. This electrolytic solution provided ions to the electrodes during oxidation and reduction of the redox couple.

## Results and Discussions

The Au catalyst layers were activated by different cleaning procedures, and the NWs were grown as described in the previous section and inspected by an SEM. We define a figure of merit (FoM) using Eq. 4 to determine the optimal activation method in terms of the crystalline quality and alignment of the grown NWs and the number of NWs per sample area (i.e., sample density); the FoM value increases as the morphological quality of the NWs improves:

4 $$ \mathrm{F}\mathrm{o}\mathrm{M}=\frac{h}{\phi}\frac{\mathrm{VAF}}{\upsigma_h{\upsigma}_{\phi }{\upsigma}_{\mathrm{VAF}}}\rho, $$

where *ρ* is the sample density, *h* and *ϕ* are the mean values of the NW height and diameter, respectively, and *σ*_*h*_ and *σ*_*ϕ*_ are their respective standard deviations. VAF is the vertical alignment factor defined in Eq. 5, and *σ*_VAF_ is its respective standard deviation.

5 $$ \mathrm{V}\mathrm{A}\mathrm{F}={\left(1+\frac{{\left(\theta -90{}^{\circ}\right)}^2}{90{}^{\circ}}\right)}^{-1}, $$

where *θ* is the mean value of the NW inclination (i.e., the angle formed by the NWs with respect to the substrate, in degrees).

The results obtained from the 11 activation variations explored in this work are summarized in Table 1. Based on the summarized data, three activation procedures that resulted in higher NW densities and FoM values were selected to be studied in further detail using other characterization techniques. The three activation solutions that we selected were H_2_O_2_:KOH [1:2], H_2_O_2_:KOH [1:3], and H_2_O_2_:NaOH [1:2], with a reaction time of 10 min. Detailed histograms of the diameter and height measurements are shown in Additional file 1: Figures S2 and S3, respectively.

**Table 1:** Summary of the activation processes utilized in this work

Activation solution	Activation time (min)	NW height, *h* (μm)	NW diameter, *ϕ* (nm)	NW inclination, *θ* (°)	Vertical alignment factor, VAF	Density (no. of NWs per 100 μm^2^)	Figure of merit, FoM
H_2_O_2_:KOH [1:2]	5	6.77 ± 1.47	0.62 ± 0.25	85 ± 26	0.41 ± 0.36	7	201
	10	4.26 ± 0.37	0.41 ± 0.11	90 ± 11	0.63 ± 0.28	140	80,257
H_2_O_2_:KOH [1:3]	5	5.09 ± 0.96	0.62 ± 0.23	88 ± 27	0.53 ± 0.29	19	1278
	10	4.59 ± 0.56	0.39 ± 0.06	88 ± 10	0.65 ± 0.28	124	98,844
H_2_O_2_:NaOH [1:2]	5	7.19 ± 1.06	0.91 ± 0.25	90 ± 17	0.60 ± 0.35	3	145
	10	4.37 ± 0.57	0.36 ± 0.08	91 ± 8	0.78 ± 0.25	172	140,393
H_2_O_2_:NaOH [1:3]	5	5.90 ± 1.19	0.53 ± 0.26	87 ± 13	0.60 ± 0.30	12	821
	10	6.02 ± 0.81	1.00 ± 0.18	96 ± 14	0.55 ± 0.31	46	3358
HNO_3_	1	4.48 ± 0.75	0.39 ± 0.10	86 ± 25	0.56 ± 0.32	14	3703
	3	5.36 ± 1.09	0.55 ± 0.19	96 ± 20	0.54 ± 0.32	24	1888
	10	5.19 ± 0.81	0.55 ± 0.15	90 ± 18	0.51 ± 0.28	12	1627

**Fig. 2:**
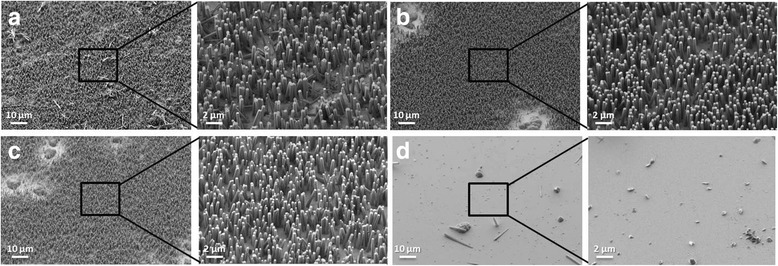
SEM images of resulting grown NWs. SEM images of 1-year-old substrates after activating for 10 min with solutions of **a** H_2_O_2_:KOH [1:2], **b** H_2_O_2_:KOH [1:3], **c** H_2_O_2_:NaOH [1:3], and **d** without any cleaning

The diverse NW densities and qualities observed in the SEM images (Fig. 2) indicate a strong dependence of these parameters on surface activation. The effect of the activation time on the resulting NW density was especially important. Figure 2a–c corresponds to the SEM images of the resulting NWs after the three preferred activation processes were carried out. The resulting ZnO NWs were of outstanding quality with high density, good uniformity, and vertical alignment. In addition, Fig. 2d shows almost complete absence of nanowires on an uncleaned 1-year-old substrate. These results, summarized in Table 2, clearly demonstrate that surface activation is an essential conditioning procedure when using gold-seeded substrates. A set of SEM images, which were used to measure the NW physical parameters, corresponding to the different activation processes based on KOH, NaOH, and HNO_3_ are shown in Additional file 1: Figures S4, S5, and S6, respectively. In addition, low magnification images from the same samples shown in Fig. 2 are shown in Additional file 1: Figure S7. It can be observed how the coverage ratio and uniformity of the growth increase with the activation methods.

**Table 2:** Summary of the results of SEM images, AFM topographies, and CV responses

Activation process	Δ*E*_p_ (mV)	Roughness (nm)	Density (no. of NWs per 100 μm^2^)	Figure of merit, FoM
H_2_O_2_:KOH [1:2] for 10 min	122	3.53 ± 0.33	140	80,257
H_2_O_2_:KOH [1:3] for 10 min	102	3.36 ± 0.18	124	98,844
H_2_O_2_:NaOH [1:2] for 10 min	112	3.01 ± 0.36	172	140,393
2-month-old, uncleaned	–	2.87 ± 0.28	0	–
1-year-old, uncleaned	–	2.74 ± 0.34	0	–

**Fig. 3:**
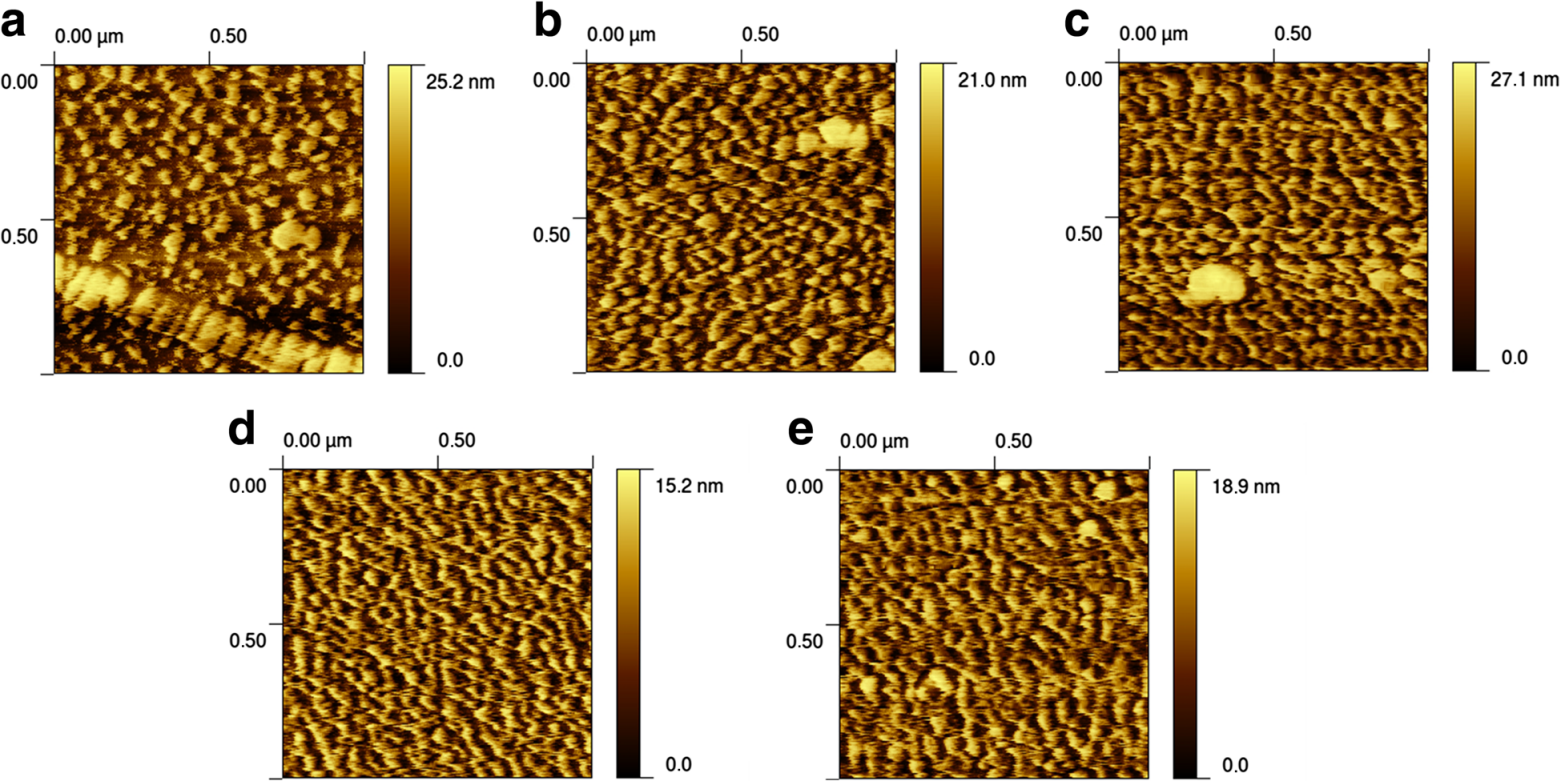
AFM images of sample surfaces. AFM images of the surface after 10-min activation by different cleaning solutions: **a** H_2_O_2_:KOH [1:2], **b** H_2_O_2_:KOH [1:3], **c** H_2_O_2_:NaOH [1:2]. Images of sample surfaces before cleaning: **d** uncleaned, 1-year-old gold layer; **e** uncleaned, 2-month-old gold layer

To study the effect of the cleaning processes on the surface roughness, the topography of the samples was measured by AFM before and after the cleaning processes (Table 2), and the obtained images of different samples are shown in Fig. 3. The values calculated for the root-mean-square (RMS) roughness show that the cleaning procedures slightly increased the surface roughness when compared to the uncleaned samples. However, the small difference in roughness, for instance, from 2.87 ± 0.28 nm for an uncleaned, 2-month-old sample to 3.53 ± 0.33 nm for the samples cleaned for 10 min using H_2_O_2_:KOH [1:2] and [1:3], is not reflective of any over-activation or other surface-damaging effects. In addition, passivation of the substrate after a long period resulted in a slightly flatter surface (from 2.87 ± 0.28 nm to 2.74 ± 0.34 nm for the uncleaned, 2-year-old sample).

On the other hand, cyclic voltammetry evaluates the activation of a gold electrode by taking into account the potential difference between the cathodic (*E*_pc_) and anodic (*E*_pa_) peak currents (Δ*E*_p_) (Fig. 1a). The potential difference should theoretically be Δ*E*_p_ = 58 mV for single-electron transfer reactions, such as the reaction between the [Fe(CN)_6_]^3−^–[Fe(CN)_6_]^4−^ redox couple and a perfect gold surface. Any increase of this value is normally interpreted as an undesired effect caused by surface contaminations.

**Fig. 4:**
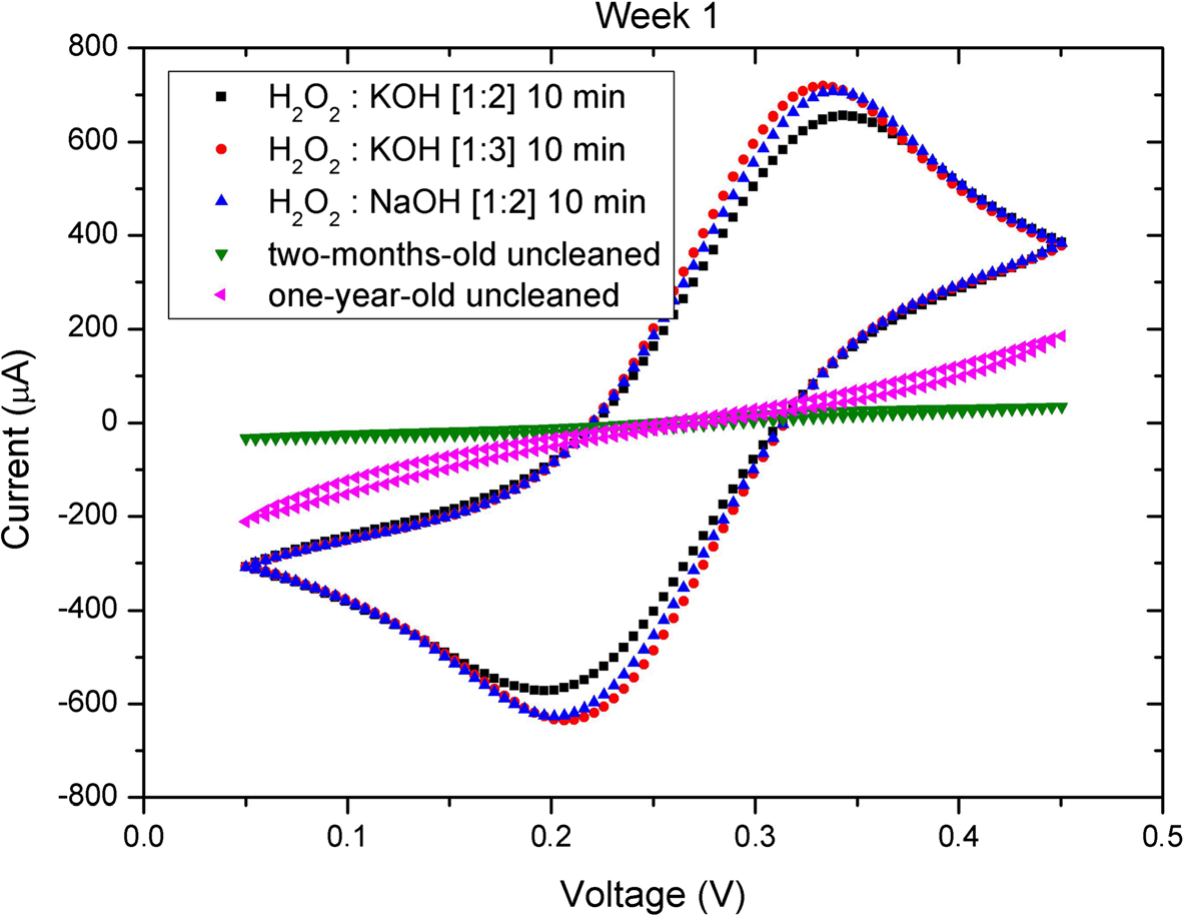
Cyclic voltammetry corresponding to different cleaning methods. Cyclic voltammetry of electrochemical cell consisting of an Ag/AgCl reference electrode, a Pt counter electrode, and different gold working electrodes activated by different cleaning processes

Cyclic voltammograms of the samples cleaned by the three different selected activation processes and two additional uncleaned samples (2-month-old and 1-year-old) are shown in Fig. 4. The distance between the redox peaks of Fe is correlated with an efficient activation process (i.e., a high FoM): the higher the FoM value, the lower the distance between the peaks (*∆E*_p_). In addition, the non-activated substrates showed poor response in terms of peak distances, and no redox peaks were observed for the 1-year-old sample. Therefore, these CV measurements can be used as a very useful indicator of the seed-layer quality for subsequent NW growth. A summary of the most important parameters associated with the results of each activation procedure is described in Table 2.

**Fig. 5:**
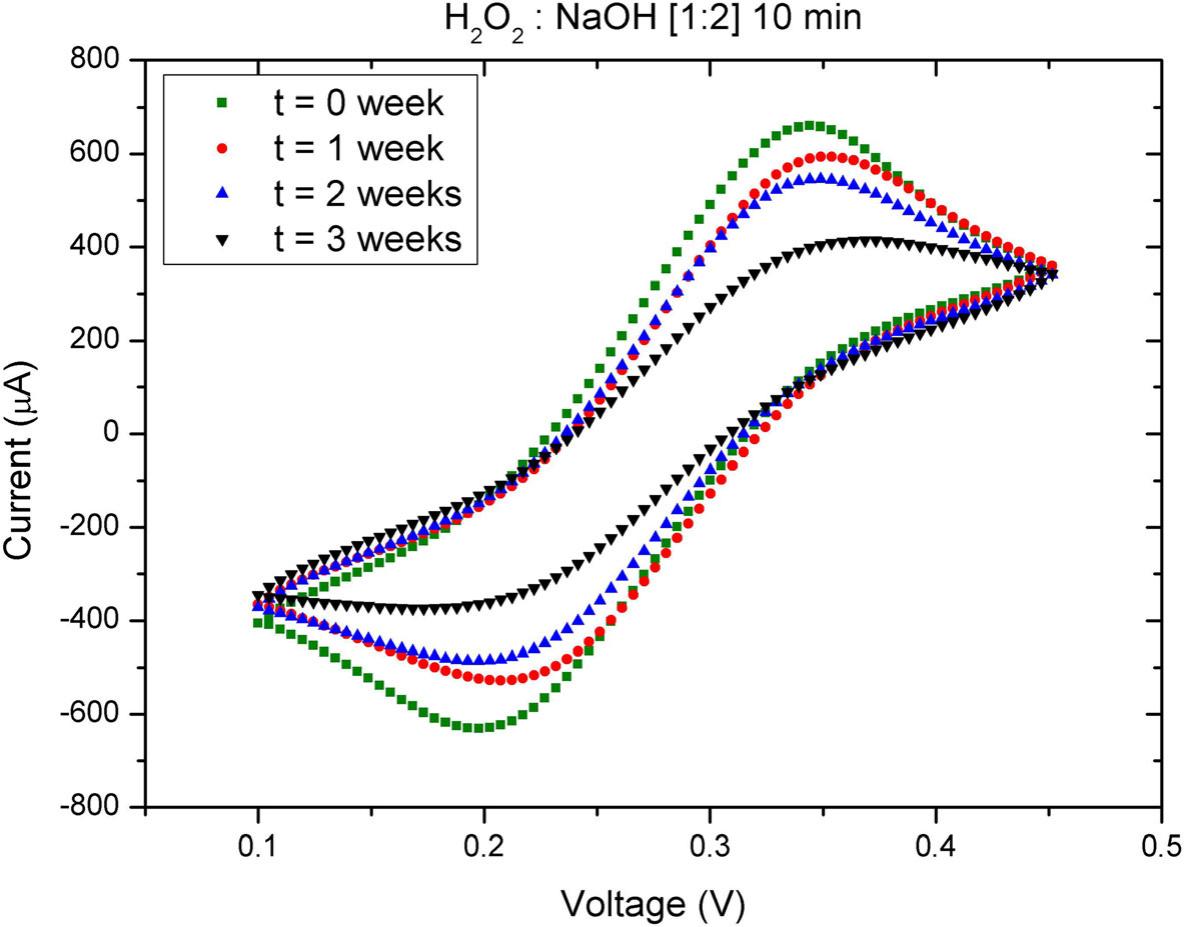
Cyclic voltammetry evolution along time. Cyclic voltammograms measured once a week for 3 weeks after an initial 10-min activation with a solution of H_2_O_2_:NaOH [1:2]

**Fig. 6:**
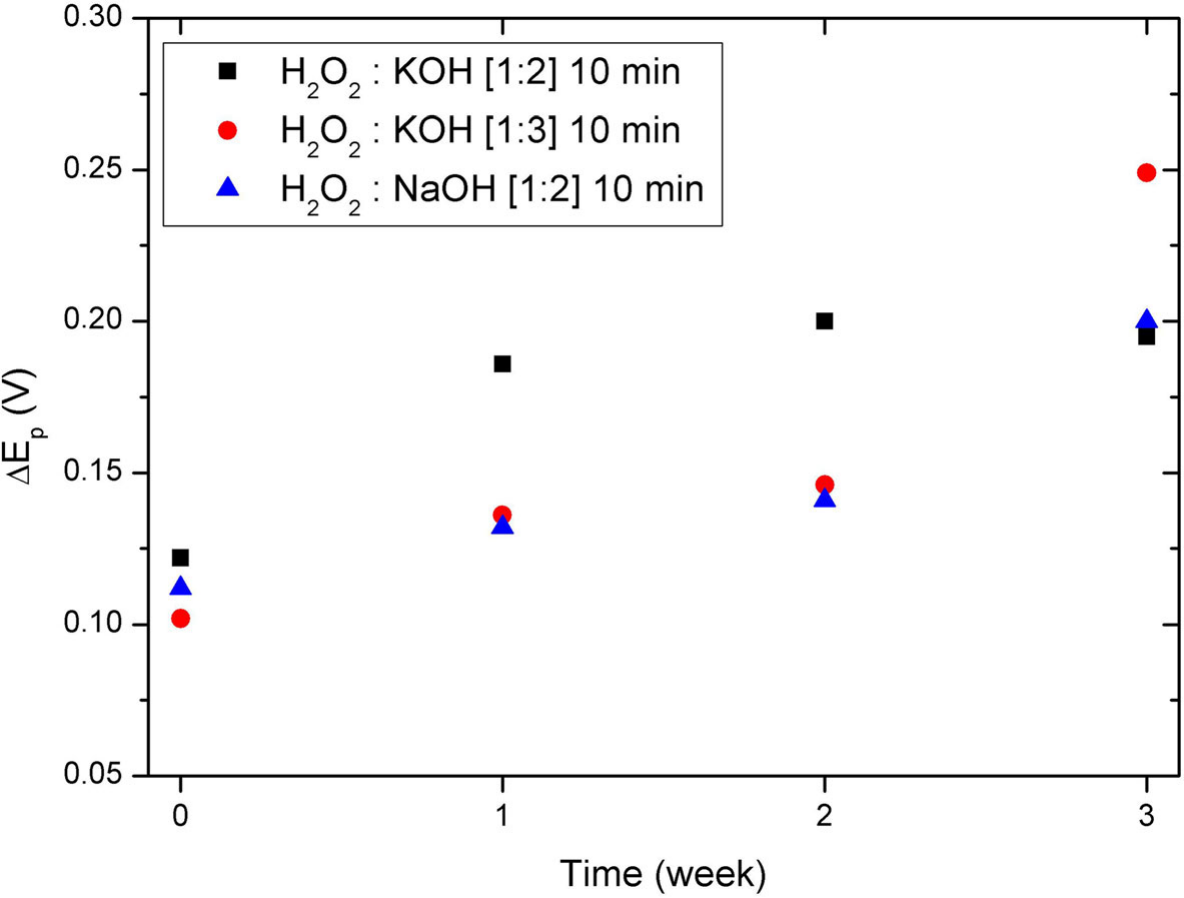
Potential difference ΔEp for different activation processes. Potential difference between the cathodic and anodic peak currents (Δ*E*_p_) measured right after activation and 1, 2, and 3 weeks later for the different activation processes

Finally, the degradation of the gold layer over time was evaluated from the CV responses. Figure 5 illustrates the cyclic voltammograms measured right after the 10-min activation of a sample with the H_2_O_2_:NaOH [1:2] solution and 1, 2, and 3 weeks later. Table 2 shows the values of Δ*E*_p_ at different measurement times for the different activation procedures. Similar characterizations of the cleaning processes based on H_2_O_2_:KOH [1:2] and H_2_O_2_:KOH [1:3] are shown in Additional file 1: Figure S8. Also, similar to Fig. 4, Additional file 1: Figure S9 shows week-by-week voltammograms corresponding to all the activation processes used. The results indicate a visible degradation of the gold electrode’s response over time, which suggests that there was accumulated contamination on the surface. Identical characterizations were performed for the other two selected cleaning procedures. The measured values of Δ*E*_p_ are compiled in Fig. 6, which shows similar degradation in CV response for all of the samples.

## Conclusions

In this work, the quality of NWs grown by a hydrothermal method was investigated for various activation treatments of the Au catalyst layer. The quality of the gold catalyst layer played an important role in achieving NWs with high quality, good uniformity, and controllable sizes. Among various activation processes tested for the Au catalyst layer, we selected three for further characterization using physical and electrochemical techniques that included AFM, SEM, and CV. The choice of the three activation processes was based on the quality of the resulting NW growth; these processes utilized three different solutions of H_2_O_2_:KOH [1:2], H_2_O_2_:KOH [1:3], and H_2_O_2_:NaOH [1:2], and they only required a cleaning duration of 10 min. Therefore, the use of these activation processes is a simple but very effective way to increase the quality and uniformity of the hydrothermally grown NWs. Moreover, the CV measurements can be used as an indicator of the seed-layer quality for subsequent NW growth. We propose establishing this technique as a standard procedure prior to ZnO NW hydrothermal growth as it is a very useful quality indicator and increases the growth reproducibility, which is especially important when working at wafer level.

## Additional file

Additional file 1: Supporting Information. (PDF 1656 kb)

## Abbreviations

NW: Nanowire: AFM, atomic force microscopy
SEM: Scanning electron microscopy
CV: Cyclic voltammogram
FoM: Figure of merit
HMTA: Hexamethylenetetramine
IPA: Isopropyl alcohol
RMS: Root-mean-square
VAF: Vertical alignment factor
