# Supplementary material for: Improving Morphological Quality and Uniformity of Hydrothermally Grown ZnO Nanowires by Surface Activation of Catalyst Layer
Source: Nanoscale Res Lett. 2017 Jan 18;12:51. doi: 10.1186/s11671-017-1838-x (PMC5241599; doi:10.1186/s11671-017-1838-x)
Supplement: Additional file 1: — Supporting Information. (PDF 1656 kb) [file 11671_2017_1838_MOESM1_ESM.pdf]

SUPPORTING INFORMATION

Improving morphological quality and uniformity of hydrothermally-grown ZnO nanowires by surface activation of catalyst layer

Gonzalo Murillo[1], Helena Lozano[1], Joana Cases-Utrera[1], Minbaek Lee[2] and Jaume Esteve[1]*

[1]Department of Nano and Microsystems, Instituto de Microelectrónica de Barcelona (IMB-CNM, CSIC), Bellaterra 08193, Spain

[2]Department of Physics, Inha University, Incheon 22212, South Korea

* Corresponding author: E-mail: gonzalo.murillo@csic.es (Dr. Gonzalo Murillo)

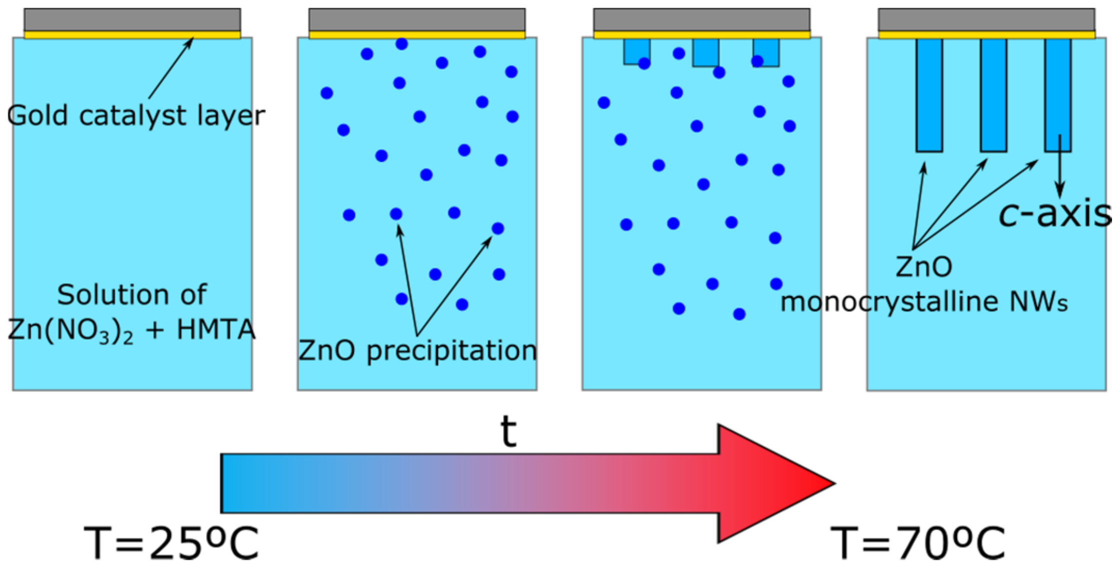

Figure S1. Schematic drawing to explain the hydrothermal method used to grow ZnO NWs.

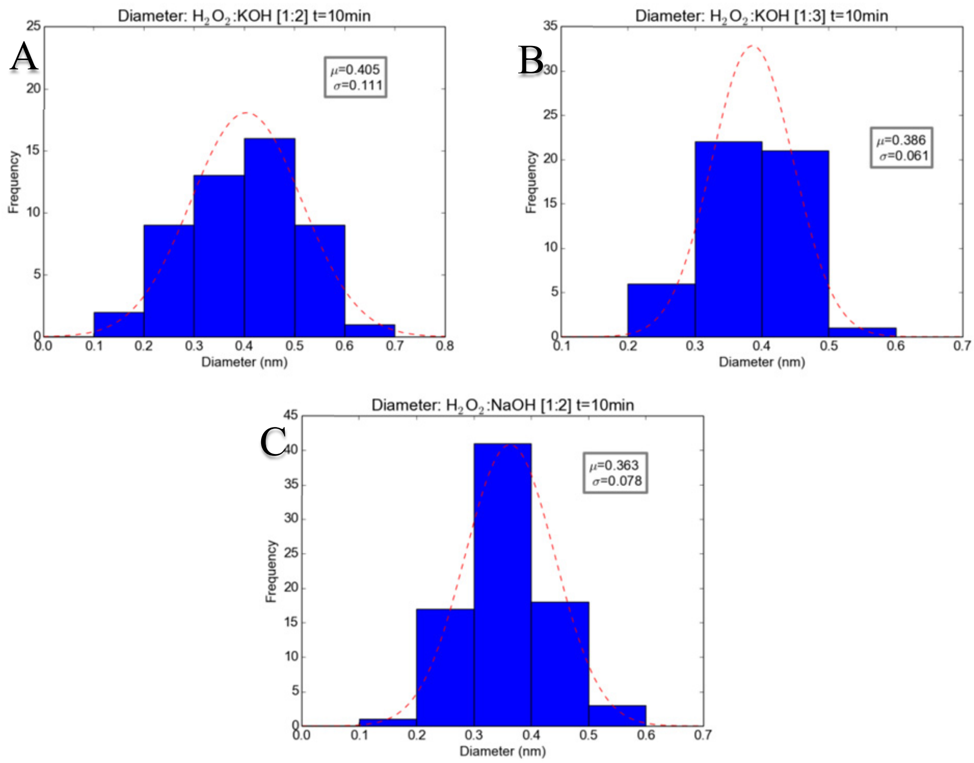

Figure S2. Histograms of the grown NW diameter values after a 10-min activation process by using several cleaning solutions: (a) H_2O_2:KOH [1:2], (b) H_2O_2:KOH [1:3], and (c) H_2O_2:NaOH [1:2].

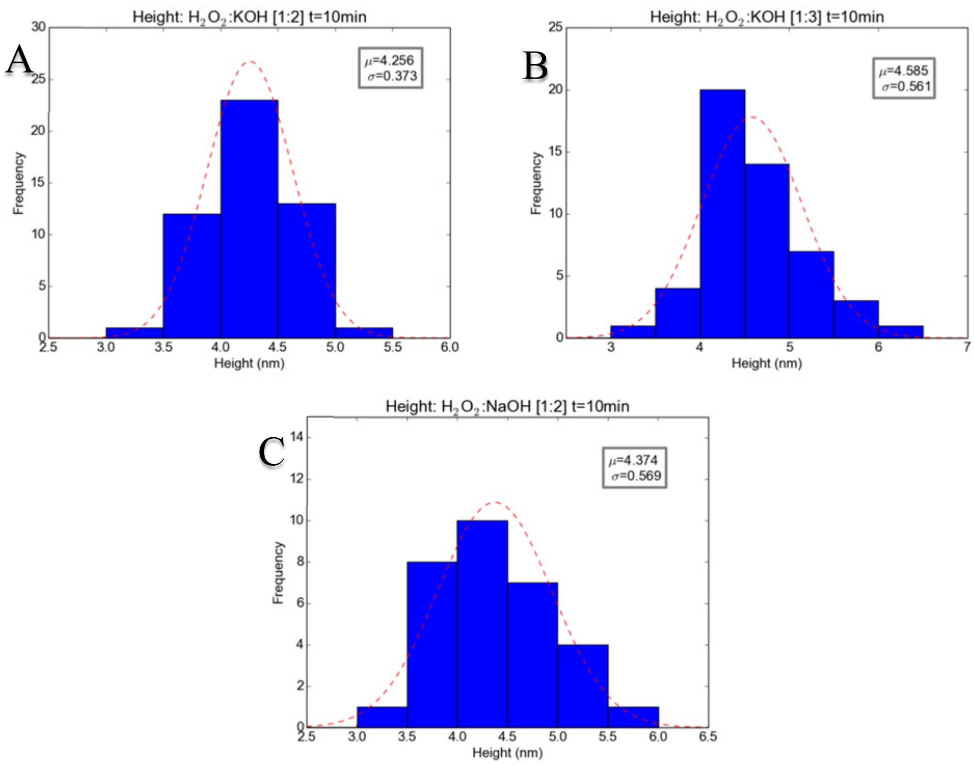

Figure S3. Histograms of the grown NW height values after a 10-min activation process by using several cleaning solutions: (a) H_2O_2:KOH [1:2], (b) H_2O_2:KOH [1:3], and (c) H_2O_2:NaOH [1:2].

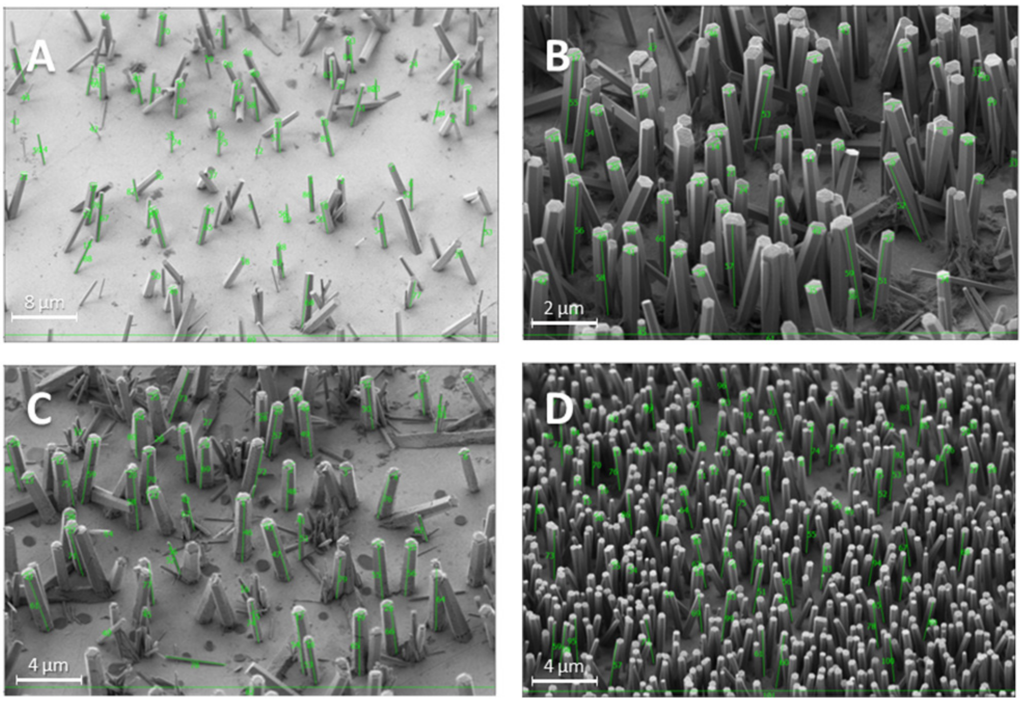

Figure S4. SEM images indicating the NW measured after a cleaning process by using several cleaning solutions of (a) H_2O_2:KOH [1:2] for 5 min, (b) H_2O_2:KOH [1:2] for 10 min, (c) H_2O_2:KOH [1:3] for 5 min and (d) H_2O_2:KOH [1:3] for 10 min.

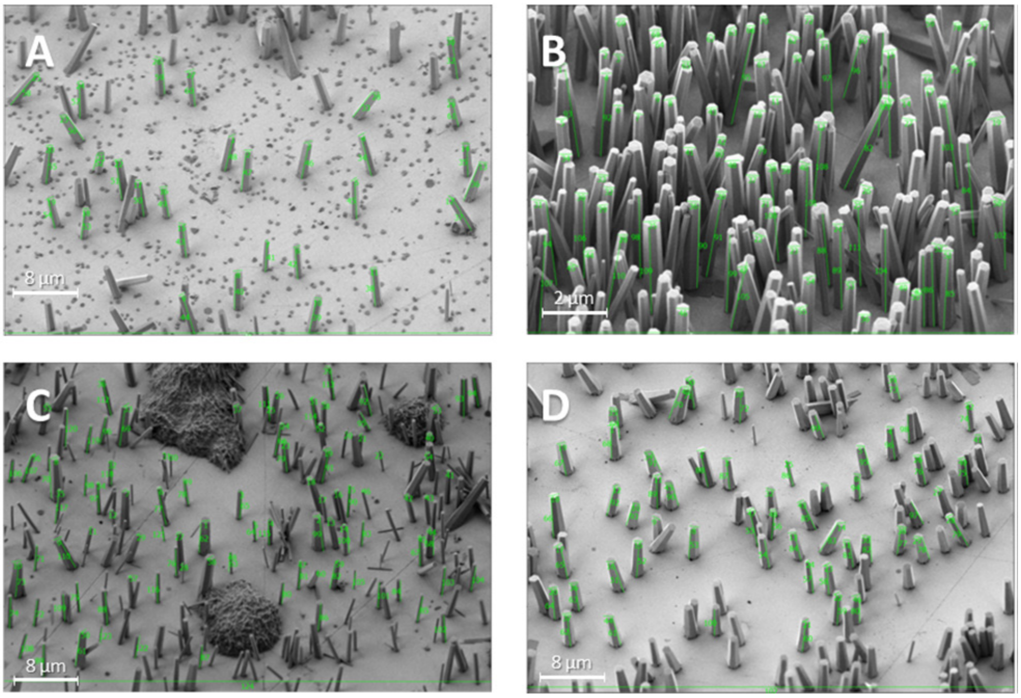

Figure S5. SEM images indicating the NW measured after a cleaning process by using several cleaning solutions: (a) H_2O_2:NaOH [1:2] for 5 min, (b) H_2O_2: NaOH [1:2] for 10 min, (c) H_2O_2: NaOH [1:3] for 5 min and (d) H_2O_2: NaOH [1:3] for 10 min.

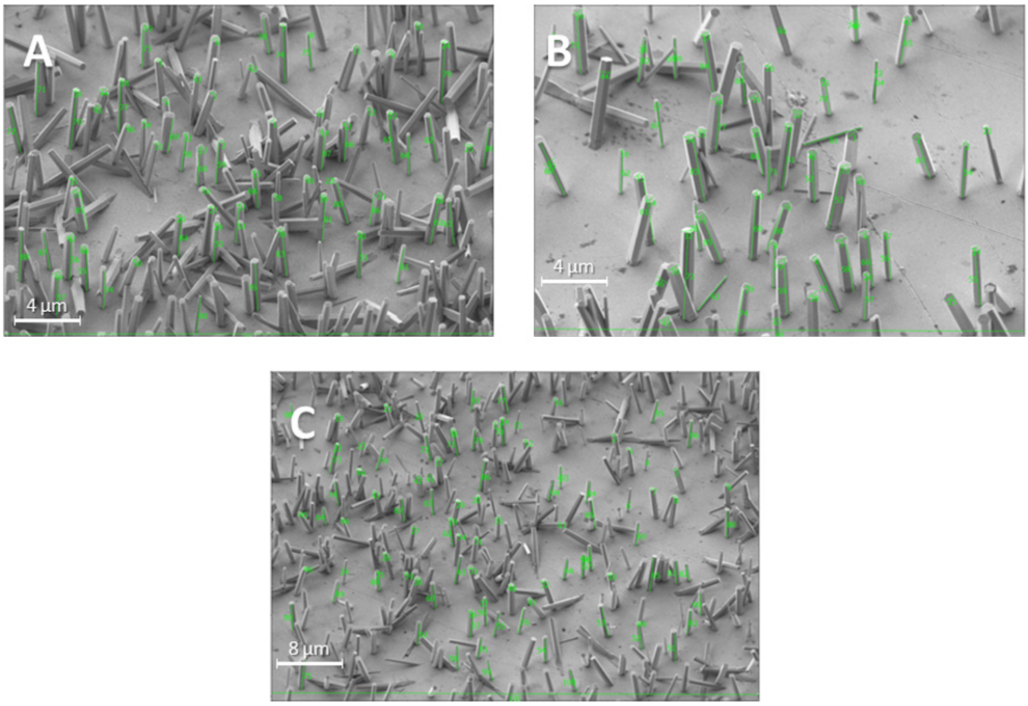

Figure S6. SEM images indicating the NW measured after a cleaning process based on HNO_3 for (a) 1 min, (b) 3 min and (c) 10 min.

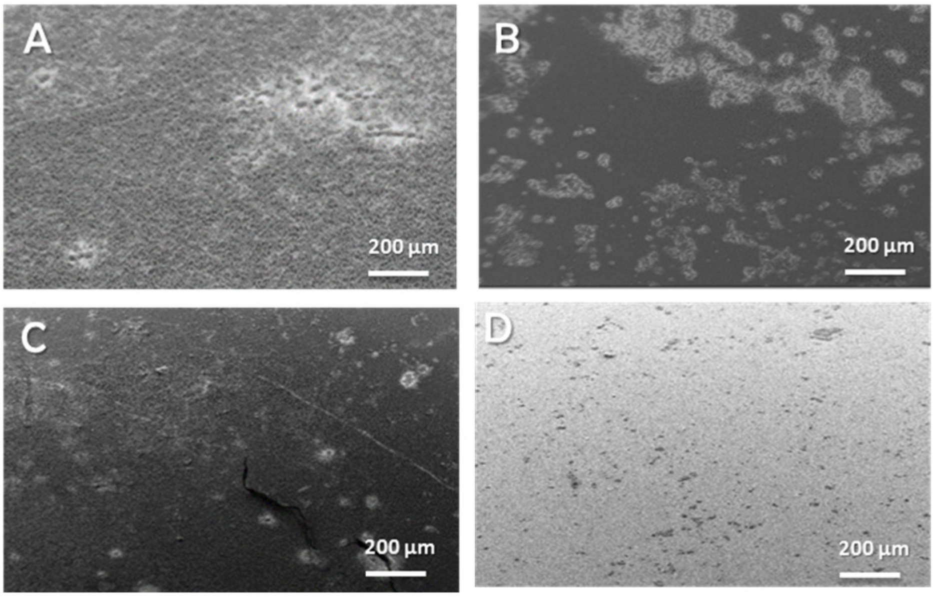

Figure S7. Low-magnification SEM images of one-year-old substrates after activating for 10 min with solutions of (a) H_2O_2:KOH [1:2], (b) H_2O_2:KOH [1:3], and (c) H_2O_2:NaOH [1:3], and (d) without any cleaning.

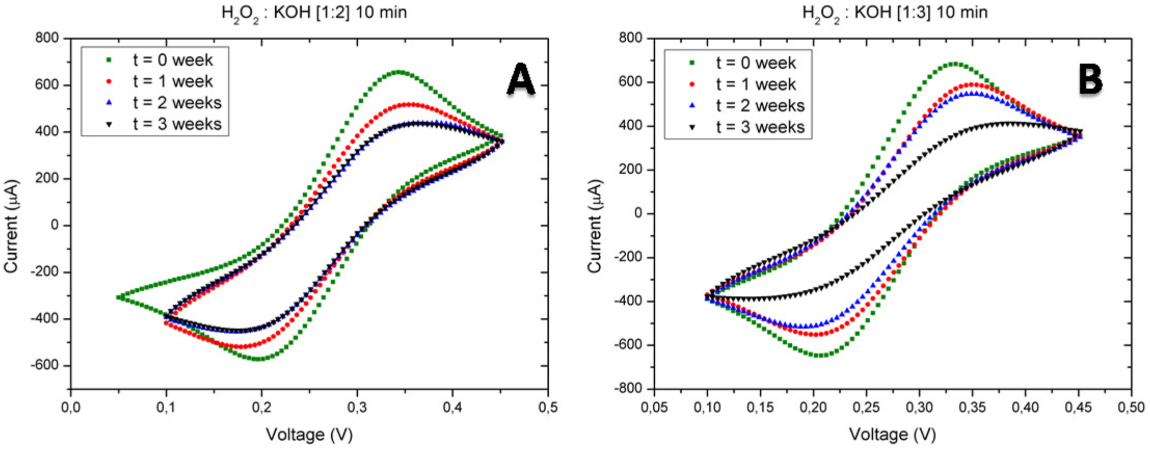

Figure S8. Cyclic voltammograms corresponding to activation process based on a 10-min cleaning with a two different solutions: (a) H_2O_2:KOH [1:2] and (b) H_2O_2:KOH [1:3], measured right after performing the activation step and one, two or three weeks later.

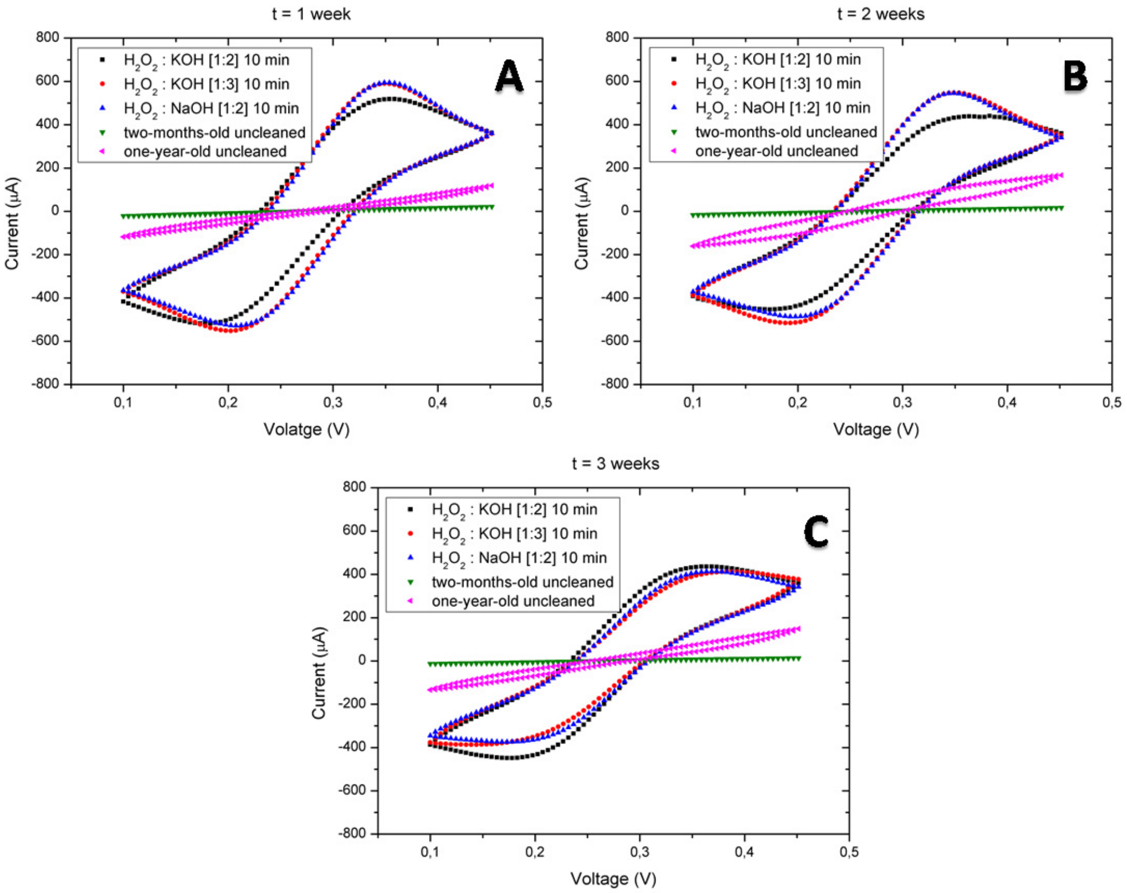

Figure S9. Cyclic voltammograms of all cleaning methods (a) one week, (b) two weeks or (c) three weeks after performing the different activation processes.
